# Supplementary figures and images for: ELISA detection of MPO-DNA complexes in human plasma is error-prone and yields limited information on neutrophil extracellular traps formed in vivo
Source: PLoS One. 2021 Apr 22;16(4):e0250265. doi: 10.1371/journal.pone.0250265 (PMC8062102; doi:10.1371/journal.pone.0250265)

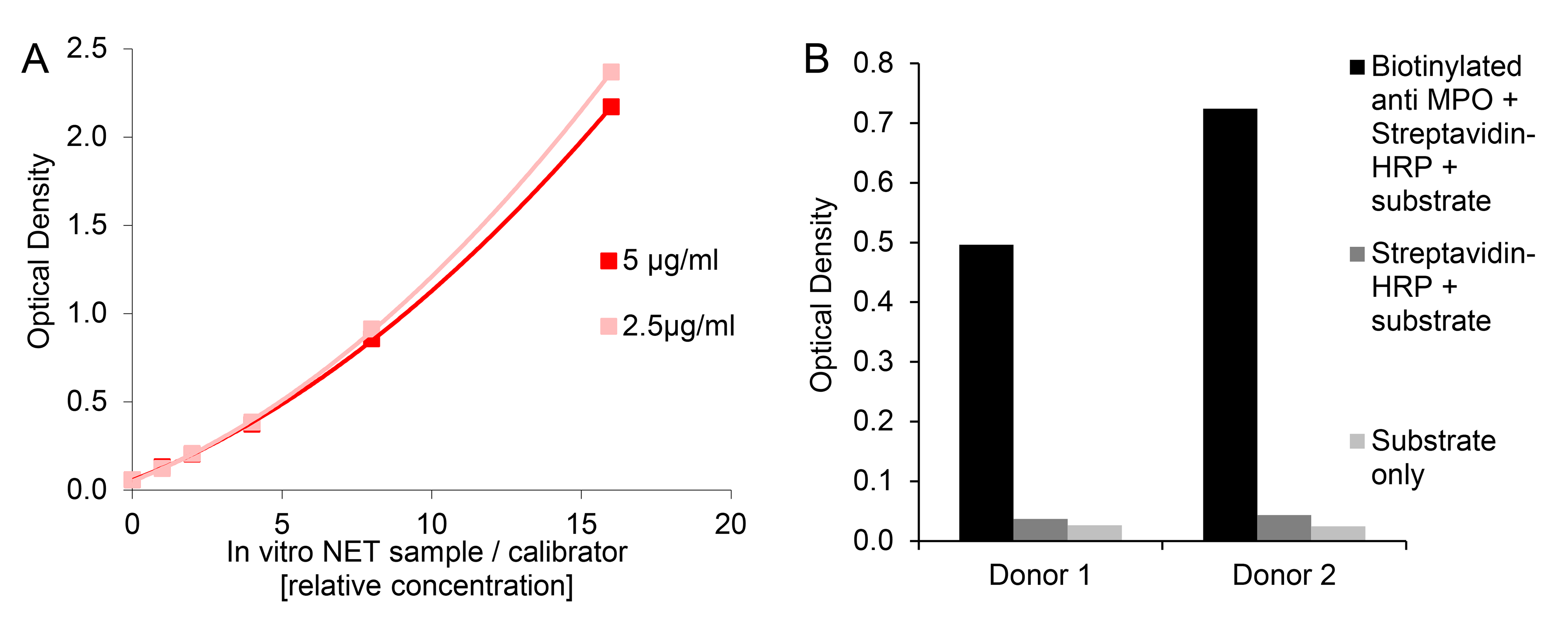

Supplement: S1 Fig — A) Microplate wells were coated with 5 μg/ml or 2.5 μg/ml MPO antibody. Supernatant of PMA-treated neutrophils was diluted (two-fold dilution series) and applied for assessment of MPO-DNA complexes using the initial ELISA protocol. Preparation of calibrator batch #1 was used in this experiment. B) Microwells were left uncoated, were blocked and then incubated with 1:2 diluted plasma samples. For signal development, a complete detection system consisting of 0.5 μg/ml biotinylated mouse anti-human MPO monoclonal antibody (no. HM2164BT, HyCult Biotech), 0.5 μg/ml streptavidin-HRP and ABTS substrate solution was sequentially applied. Where indicated, the biotinylated MPO antibody or additionally the streptavidin-HRP conjugate were omitted. (TIF) [file pone.0250265.s001.tif]

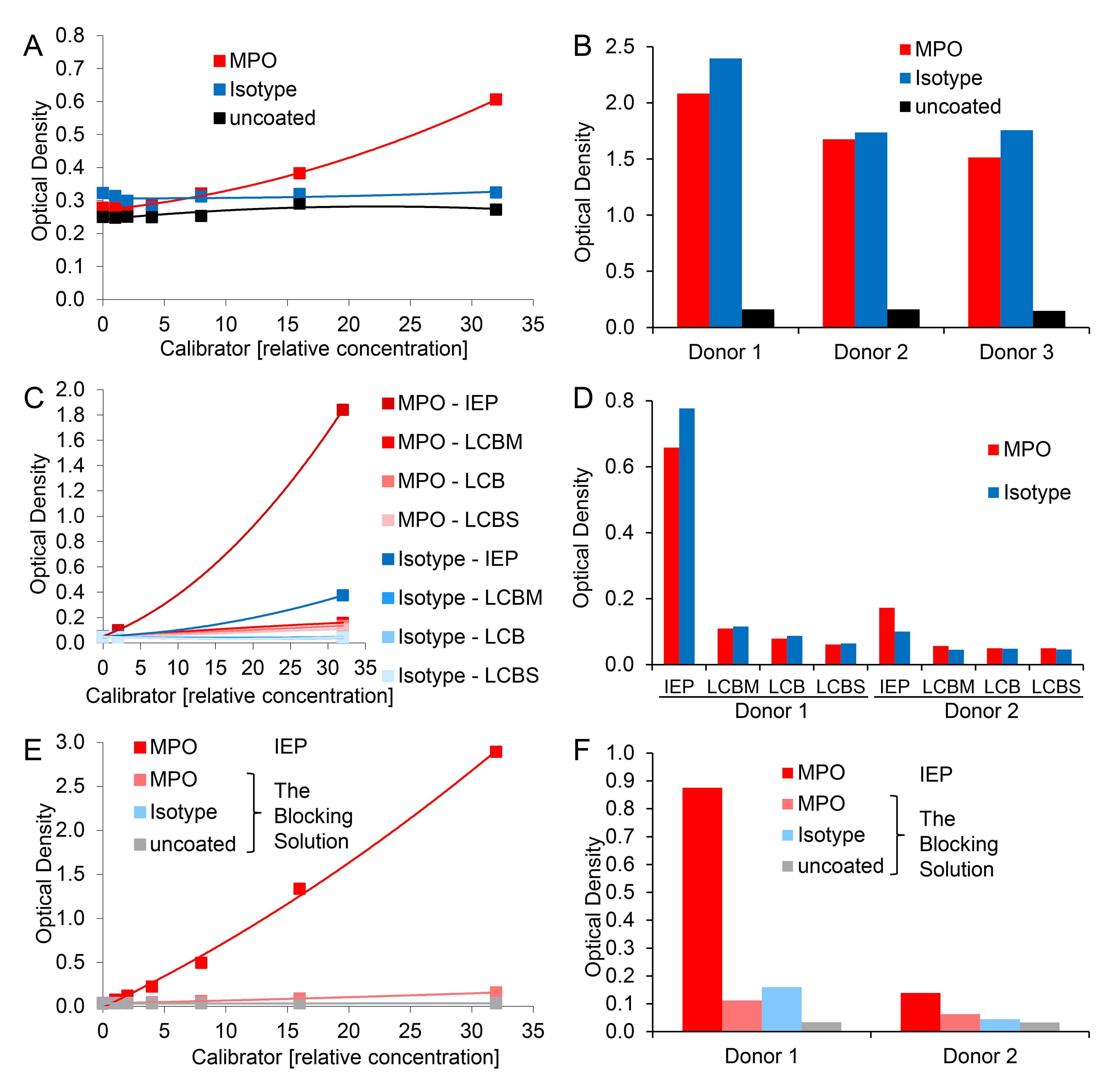

Supplement: S2 Fig — A) and B) Microwells were coated with MPO antibody, isotype control or were left uncoated and incubated with A) calibrator or B) 1:4 diluted plasma samples. The assay was performed according to the initial MPO-DNA complex ELISA protocol, but blocking buffer, sample diluent and detection antibody diluent were replaced by an in-house blocking buffer. C) and D) Microwells were coated with MPO antibody or isotype control. C) Calibrator or D) 1:5 diluted plasma samples were assayed using different sample and detection antibody diluents: IEP, diluents as outlined for the initial ELISA protocol; LCBM, LowCross-Buffer Mild; LCB, LowCross-Buffer; LCBS, LowCross-Buffer Strong. E and F) Microwells were coated with 5 μg/ml MPO antibody, isotype control or were left uncoated and incubated with E) calibrator or F) 1:5 diluted plasma samples using different reagents: for samples labeled “IEP”, blocking buffer as well as sample diluent and detection antibody diluent were applied as outlined for the initial MPO-DNA complex ELISA protocol whereas for samples labeled “The Blocking Solution”, The Blocking Solution (Candor Bioscience) was used for blocking, sample dilution and detection antibody dilution. Note that data points for isotype control in E) are not visible since they presented with almost identical optical densities as for uncoated wells. (TIF) [file pone.0250265.s002.tif]

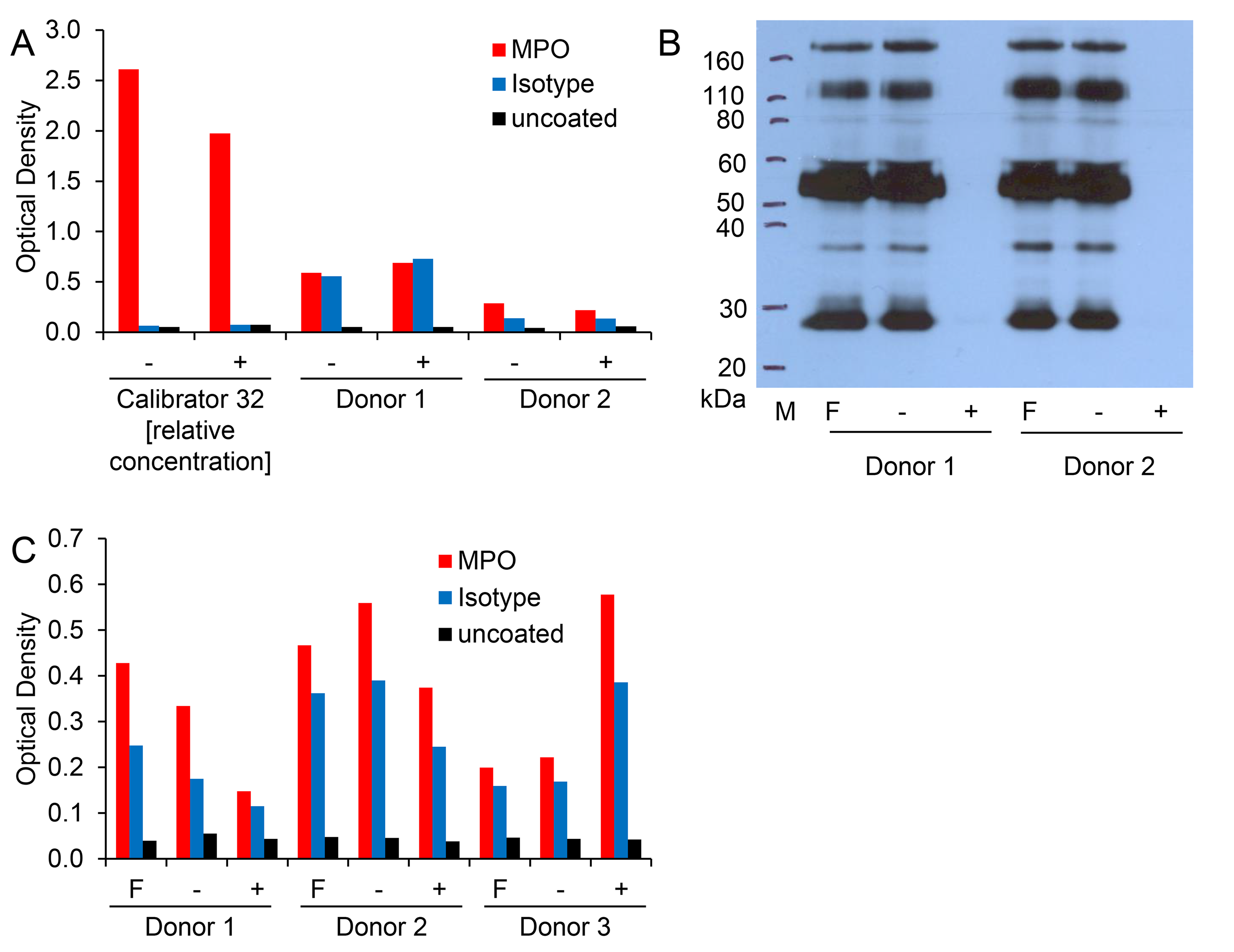

Supplement: S3 Fig — A) Calibrator (at a relative concentration of 32) and plasma samples (at 1:2 dilution) were pre-adsorbed (+) or not (-) on isotype control coated and blocked microwells for 2 h. Samples were then transferred onto microwells coated with MPO antibody, isotype control or left uncoated and processed as indicated for the initial MPO-DNA complex ELISA protocol. B) and C) Plasma immunoglobulins were removed with protein A/G PLUS-agarose. Precleared plasma (+), samples comparably incubated without protein A/G (-) or fresh plasma (F) were then subjected to B) Western blotting or C) the initial MPO-DNA complex ELISA protocol using MPO antibody, isotype control or uncoated microwells. For ELISA, a final plasma dilution of 1:4 was applied. Expected molecular weights are: IgG heavy chains: 50 kDa (IgG1, IgG2 and IgG4) and 60 kDa (IgG3); IgM heavy chains: 70 kDa; IgG and IgM light chains: 23 kDa. M, molecular weight marker (Sharp Pre-stained Protein Standard, given in kDa). (TIF) [file pone.0250265.s003.tif]

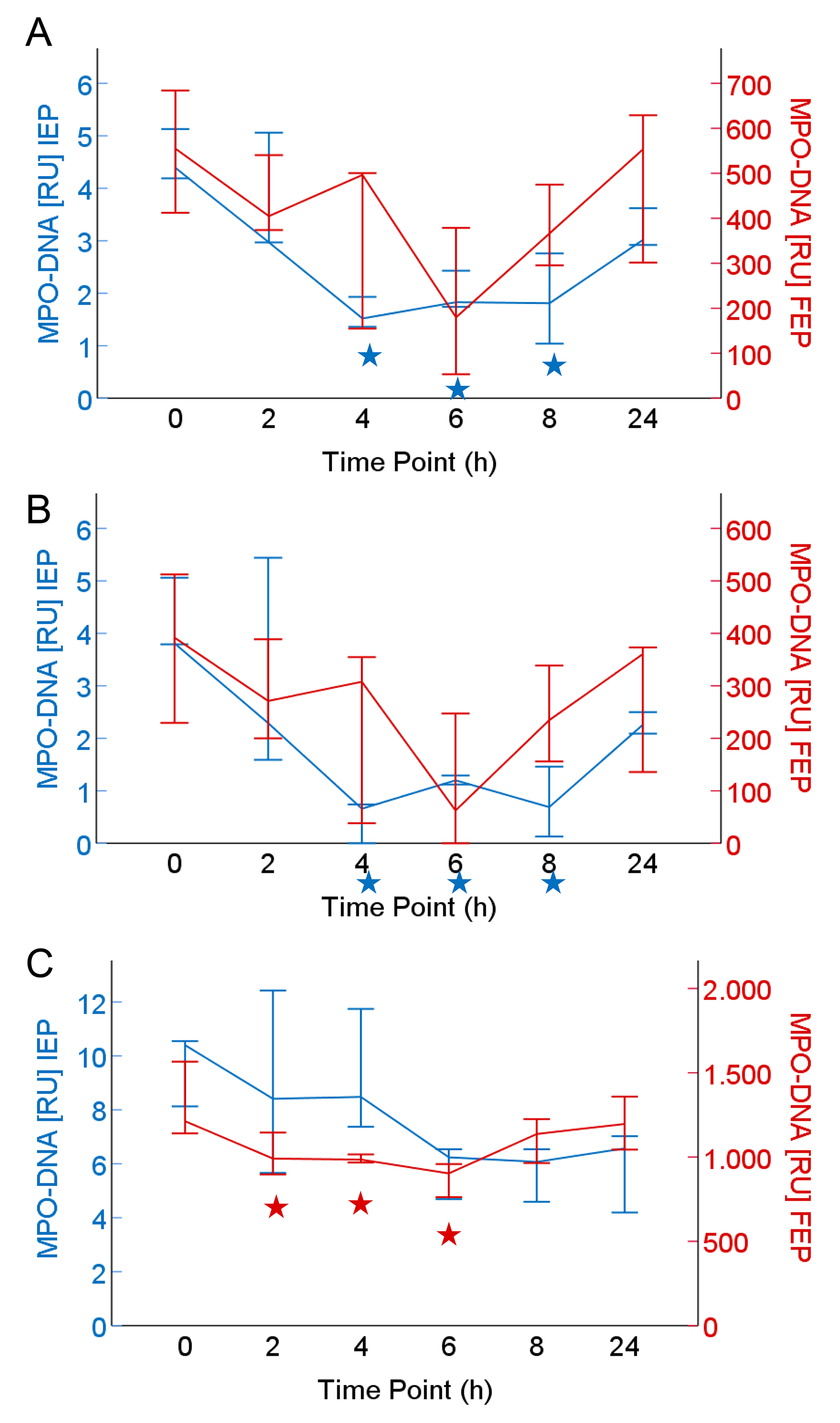

Supplement: S4 Fig — Plasma samples of seven healthy volunteers at baseline (0 h) and at various time points after infusion of 2 ng/kg LPS were assessed for MPO-DNA complexes according to the initial (IEP) as well as the final, modified ELISA protocol (FEP). A) MPO-DNA concentrations were determined as outlined in materials and methods, i.e. the optical density obtained for the isotype control was first subtracted from the optical density of MPO antibody-coated wells. The resulting value was then used to calculate the MPO-DNA complex content according to the calibration curve. B) Additionally, an alternative calculation method was applied, since calibrator curves were non-linear with the inherent problem that net signal excerpted from a low relative concentration segment in the calibration curve would correspond to a greater concentration difference (between actual sample and its isotype control) than the same net signal excerpted from a high relative concentration segment on the calibration curve. Thus, the optical densities obtained for the isotype control and MPO antibody-coated wells were separately used to calculate concentrations in relation to the calibration curve. Thereafter, the established value for the isotype control was subtracted from the calculated concentration of MPO-antibody coated wells. C) For comparison, calculated MPO-DNA complex values without isotype control subtraction are provided. Medians and their 50% confidence intervals are indicated. ★ p < 0.05 according to Wilcoxon signed-rank test (in comparison to baseline); RU: relative units. (TIF) [file pone.0250265.s004.tif]

Fig. 4B

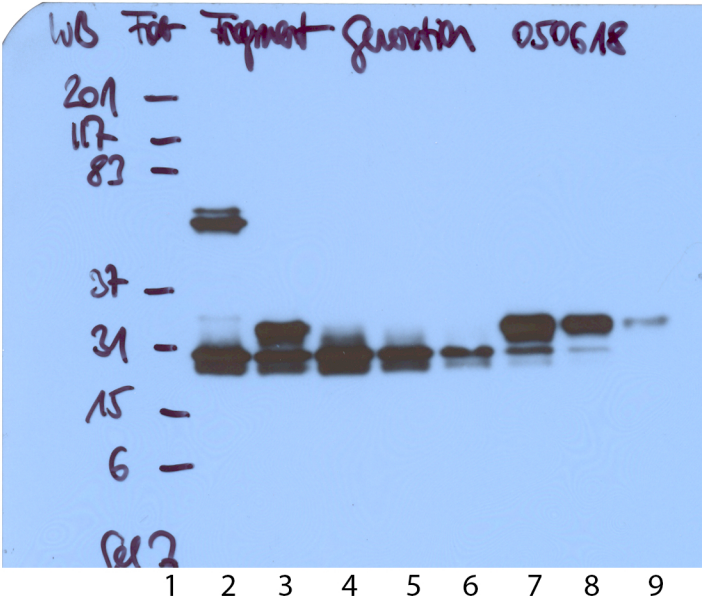

Fig. 4C

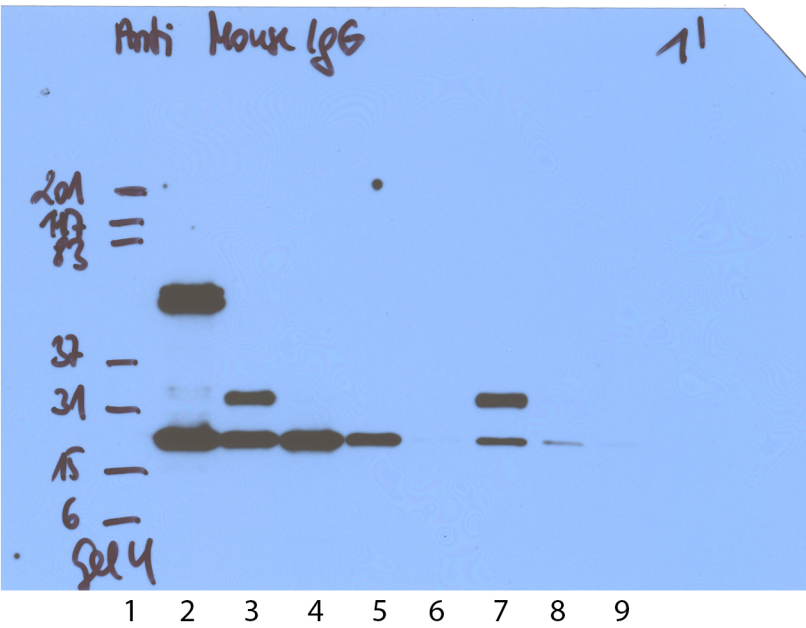

S Fig. 3B

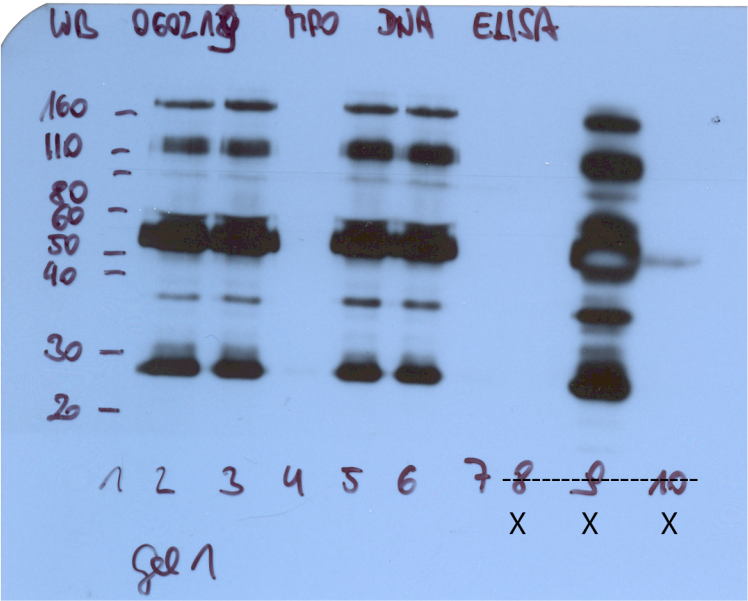

Supplement: S1 Raw images — (PDF) [file pone.0250265.s008.pdf]
