# Supplementary material for: ELISA detection of MPO-DNA complexes in human plasma is error-prone and yields limited information on neutrophil extracellular traps formed in vivo
Source: PLoS One. 2021 Apr 22;16(4):e0250265. doi: 10.1371/journal.pone.0250265 (PMC8062102; doi:10.1371/journal.pone.0250265)
Supplement: S1 Table — (DOCX) [file pone.0250265.s005.docx]

S1 Table. Concomitant analysis of control- and PMA-treated neutrophil supernatant by MPO-DNA ELISA.

| **Batch** | **Treatment** | **MPO-DNA [optical density]** | | | |
| --- | --- | --- | --- | --- | --- |
|  |  | 1:50 dilution | 1:400 dilution | 1:1600 dilution | n |
| #1 | Control | 0.065 ± 0.002 | 0.040 ± 0.007 | 0.082 ± 0.001 | 2 |
|  | PMA | 1.924 ± 1.578 | 0.147 ± 0.064 | 0.060 ± 0.016 | 2 |
| #2 | Control | 0.081 ± 0.017 | 0.051 ± 0.024 | 0.045 ± 0.019 | 3 |
|  | PMA | 1.515 ± 0.193 | 0.186 ± 0.038 | 0.074 ± 0.028 | 3 |
| #3 | Control | 0.058 ± 0.022 | 0.038 ± 0.007 | 0.036 ± 0.001 | 2 |
|  | PMA | 2.389 ± 0.596 | 0.206 ± 0.004 | 0.084 ± 0.012 | 2 |
| DNA, deoxyribonuleic acid; MPO, myeloperoxidase; PMA, phorbol 12-myristate 13-acetate. | | | | | |

Neutrophils (obtained from two healthy donors: batches #1 and #2 from donor 1, batch #3 from donor 2) were stimulated with PMA or solvent control for 3 h and the resulting supernatants were concomitantly assessed within the same assay by the initial MPO-DNA ELISA protocol. Optical density range is given for sample dilutions of 1:1600, 1:400 and 1:50, corresponding to relative concentrations of 1, 4 and 32. Mean ± SD refer to the number (n=2 or 3) of assays for MPO-DNA complexes performed with the respective batch.
