# Supplementary material for: ELISA detection of MPO-DNA complexes in human plasma is error-prone and yields limited information on neutrophil extracellular traps formed in vivo
Source: PLoS One. 2021 Apr 22;16(4):e0250265. doi: 10.1371/journal.pone.0250265 (PMC8062102; doi:10.1371/journal.pone.0250265)
Supplement: S2 Table — (DOCX) [file pone.0250265.s006.docx]

S2 Table. AAA patient demographics: categorical variables

| **Characteristic** | **AAA patients (n = 40)** |
| --- | --- |
|  | *n (%)* |
| Sex |  |
| Female | 3 (7.5%) |
| Male | 37 (92.5%) |
| Smoker status |  |
| Never | 4 (10.0%) |
| Past | 15 (37.5%) |
| Current | 21 (52.5%) |
| Hypertension | 34 (85.0%) |
| Hyperlipidemia | 33 (82.5%) |
| Peripheral artery disease | 7 (17.5%) |
| Coronary heart disease | 14 (35.0%) |
| Myocardial infarction | 9 (22.5%) |
| Stroke | 2 (5.0%) |
| Diabetes mellitus | 9 (22.5%) |
| COPD | 14 (35.0%) |
| Nephropathy/renal cysts | 13 (32.5%) |
| AAA family history |  |
| No | 35 (87.5%) |
| Yes | 4 (10.0%) |
| Unknown | 1 (2.5%) |
| AAA morphology |  |
| Saccular | 9 (22.5%) |
| Fusiform | 26 (65.0%) |
| Unknown | 5 (12.5%) |
| ILT presence |  |
| No | 1 (2.5%) |
| Yes | 37 (92.5%) |
| Unknown | 2 (5.0%) |
| AAA, abdominal aortic aneurysm; COPD, chronic obstructive pulmonary disease; ILT, intraluminal thrombus. | |
