# Supplementary material for: ELISA detection of MPO-DNA complexes in human plasma is error-prone and yields limited information on neutrophil extracellular traps formed in vivo
Source: PLoS One. 2021 Apr 22;16(4):e0250265. doi: 10.1371/journal.pone.0250265 (PMC8062102; doi:10.1371/journal.pone.0250265)
Supplement: S3 Table — (DOCX) [file pone.0250265.s007.docx]

S3 Table. AAA patient demographics: metric variables

| **Characteristic** | **AAA patients (n = 40)** |
| --- | --- |
|  | *Median (IQR)* |
| Age [years] | 70.6 (10.7) |
| Body mass index [kg/m^2^] | 27.9 (5.6) |
| Maximum AAA diameter [mm] | 57.15 (11.88) |
| Aneurysm volume [cm^3^] | 145.31 (84.32) |
| Maximum ILT diameter [mm] | 21.60 (10.50) |
| ILT volume [cm^3^] | 74.69 (80.24) |
| White blood cells [G/l] | 6.40 (2.00) |
| Lymphocytes [G/l] | 1.60 (1.01) |
| Monocytes [G/l] | 0.50 (0.30) |
| Neutrophils [G/l] | 4.00 (1.53) |
| Red blood cells [T/l] | 4.55 (0.77) |
| Hemoglobin [g/dl] | 14.60 (2.13) |
| Hematocrit [%] | 41.35 (6.45) |
| Platelets [G/l] | 156.0 (85.5) |
| C-reactive protein [mg/dl] | 0.37 (0.41) |
| Fibrinogen - Clauss [mg/dl] | 396.00 (138.00) |
| D-dimer [µg/ml] | 1.32 (1.78) |
| MPO [ng/ml] | 13.32 (13.13) |
| NE [ng/ml] | 4.95 (3.33) |
| DNA-histone complexes [RU] | 26.34 (39.32) |
| CitH3 [ng/ml] | 361.57 (203.31) |
| MPO-DNA complexes [RU] | 295.53 (226.95) |
| AAA, abdominal aortic aneurysm; CitH3, citrullinated histone H3; DNA, deoxyribonucleic acid; ILT, intraluminal thrombus; IQR, interquartile range; MPO, myeloperoxidase; NE, neutrophil elastase. | |
