# Supplementary material for: ELISA detection of MPO-DNA complexes in human plasma is error-prone and yields limited information on neutrophil extracellular traps formed in vivo
Source: PLoS One. 2021 Apr 22;16(4):e0250265. doi: 10.1371/journal.pone.0250265 (PMC8062102; doi:10.1371/journal.pone.0250265)
Supplement: Attachment — Submitted filename: PONE-D-20-40740 Response to Reviewers.pdf [file pone.0250265.s010.pdf]

MEDICAL UNIVERSITY OF VIENNA

Department of General Surgery – Medical University of Vienna
Division of Vascular Surgery
Vienna General Hospital AKH 25.05.002
Lazarettgasse 14, A - 1090 Vienna, Austria

**Assoc. Prof. Christine Brostjan, Ph.D.**	
Phone	+ 43 1 40400 73514
Fax	+ 43 1 40400 73593
Email	christine.brostjan@meduniwien.ac.at

PLOS ONE
Editorial Office

March 26th, 2021

Dear Editor,
we thank you for the opportunity to submit a revised version of our manuscript PONE-D-20-40740

"ELISA detection of MPO-DNA complexes in human plasma is error-prone and yields limited information on neutrophil extracellular traps formed *in vivo*"

by Hubert Hayden, Nahla Ibrahim, Johannes Klopf, Branislav Zagrapan, Lisa-Marie Mauracher, Lena Hell, Thomas M. Hofbauer, Anna S. Ondracek, Christian Schoergenhofer, Bernd Jilma, Irene M. Lang, Ingrid Pabinger, Wolf Eilenberg, Christoph Neumayer and Christine Brostjan

We sincerely appreciate the comments and points of critique raised by the referees and have modified the manuscript accordingly. Changes are highlighted in yellow in the manuscript text and the point-by-point reply is given below. Additionally, a clean, unmarked version of the revised manuscript is provided and the editorial instructions regarding

1. PLOS ONE's style requirements have been followed.
2. The original uncropped and unadjusted images are shown in the Figures 4 and S3 and hence do not need to be posted at a public data repository.
3. The ORCID iD for the corresponding author has been validated in Editorial Manager.

Reviewer #1:
This paper reports a very comprehensive test of a standard ELISA that has been used to measure DNA-MPO complexes in patient plasma. The assays have been used in a number of published studies including some in high profile journals. The authors conclude "we would thus like to alert the NET research community that ELISA-based quantitation of MPO-DNA complexes in plasma samples may be error-prone." I would suggest that this is too mild a warning. The series of excellent experiments (briefly summarized below) actually shows that the ELISA is dominated by non-specific reactions, and is therefore completely useless for clinical or biological studies. The study also has warning for any sandwich ELISA used to measure antigens in plasma. I enthusiastically recommend publication, and I have only minor suggestions for improvement.

I would suggest perhaps more strongly condemning the assay and the published results that have used it. Also I would suggest adding at the end of the abstract the more general warning about ELISA for plasma proteins.
Reply: We would like to thank the reviewer for the very appreciative words on our work and we have tried to phrase our warning statement more explicitly (including a more general warning) in the Abstract (line 44) and Discussion section (line 709).

In Fig. 1C the high signal with the isotype Ab control is clearly noted. This suggests that the signal from plasma is completely non-specific. However it is also striking that donors 1 and 2 are 10 X different: is this real and what is its significance?
Reply: Yes, as noted by the reviewer there is substantial difference between donors in this "interfering substance/reaction" in plasma but the magnitude is reproducible when the same samples are assayed on different ELISA plates. A comparable statement is now included in the Results section (line 378).

Fig. 2 shows that different combinations of capture and detection Abs have poor detection of pure calibrator, except when MPO is detected with a two-step streptavidin. However this completely fails in diluted plasma.
Fig. 3 explores and eliminates some possible sources of non-specificity. Preadsorption did not help.
Fig. 4 shows that the non-specificity in plasma is not due to the Fc fragment, since Fab gave the same results.
Fig. 5 shows that DNAse eliminated the signal from the pure calibrator, as expected for detection by anti-DNAse, but left the large non-specific signal in plasma.
Fig. 6 is a very important experiment, where the ELISA is tested by spiking calibrator into plasma. The signal from the spiked calibrator was completely swamped in 1:4 plasma, and showed severe non-specificity even in 1:100 plasma.
Fig. 7 further explores plasma dilution, with no help.
Fig. 8 tests whether adding mouse IgG or TruBlock can improve specificity. They discovered one extreme condition where the non-specific signal from isotype Ab was small: 10% TruBlock added to 1:100 diluted plasma. Here I think it would be important to specify the concentration of calibrator, and discuss whether it is even close to the concentrations expected in 1:100 diluted plasma. Apparently not, since the assay showed no significant correlation with related markers in a large set of patient plasmas.
Reply: If we set the median MPO-DNA ELISA signal of AAA patients (296 RU) and healthy donors at baseline (555 RU) in relation to the calibrator sample generated from isolated human neutrophils (1600 RU for 2 x 10^6 neutrophils/ml), the recorded values would equal 0.4 - 0.7 x 10^6 neutrophils/ml or about 10% of blood neutrophils (normal blood count of 2 - 7.5 x 10^6/ml) stimulated for NETosis. Considering that we would expect local rather than systemic NET induction at a much lower level, the recorded MPO-DNA values seem highly unlikely. We have included a similar statement in the Discussion section (line 809).

Reviewer #2:
General Comments: This is an extremely well-designed and described analysis of the methodological limitations in quantifying the concentration of myeloperoxidase (MPO)-DNA complexes via ELISA in human plasma. Analysis of MPO-DNA in circulating blood is increasingly used as a systemic biomarker for the release of so-called neutrophil extracellular traps (NETs) from neutrophils which either accumulate at discrete vascular sites or which are activated during various coagulopathies. NETs are complexes of DNA, histones, and various neutrophil intragranule proteins (such as MPO) released via a highly regulated mode of lytic cell death. Physiological NET release has a host-protective role in the sequestration and killing of different microbial pathogens, but sterile NET release can be pathogenic. These investigators have previously described NET release as a likely pathogenic component of abdominal aortic aneurysms and thus seek to systemically evaluate the known or proposed circulating indicators of in vivo NET release. As indicated in the title and abstract, their careful analysis indicates that current ELISA-based protocols for quantifying MPO-DNA complexes in human plasma samples suffer from multiple complications and are thus an unreliable approach for evaluating NET release as a biomarker for different human vasculopathies.

Specific Comments:
1. Given its narrow and technical focus, the "readability" of the MS by non-experts would be improved by inclusion of a graphical "abstract" that illustrates:
1) the biology of MPO-DNA complex release during NETosis;
2) the setup of the routinely used sandwich ELISA for MPO-DNA; and
3) the likely actions of plasma components on limiting efficacy of the ELISA.
Reply: Thank you for the suggestion! Since PLOS ONE does not feature graphical abstracts, we have included an illustrating summary in the Discussion section (line 744) as Figure 10.

Reviewer #3:

Summary: The manuscript by Hayden et al reports on the lack of specificity of a published ELISA for quantification of myeloperoxidase (MPO)-DNA complexes in human plasma. Much or even most of the data presented is focused on determining the source of the lack of specificity and/or figuring out a way to achieve specificity in plasma samples. Ultimately the authors find a way to achieve modest assay specificity using a particular blocking buffer and then apply their modified assay to quantify MPO-DNA complexes in plasma samples from abdominal aortic aneurysm (AAA) patients, correlating these measurements with other known markers of neutrophil activation or neutrophil extracellular trap (NET) formation but ultimately finding a lack of correlation between MPO-DNA complexes and these markers.

Broad Scope Comments: Unfortunately it is not highly uncommon for bioanalytical methods developed specifically for unique applications in biomedical research to be poorly or improperly developed then reported in the literature after relatively little scrutiny and then, in the hands of others, provide uninterpretable, irreproducible or, at worst, misleading results. So it is not terribly shocking that by merely applying proper negative controls (antibody isotype controls) the authors discovered a major flaw in an ELISA that has been used by others in published biomedical research. The authors are to be congratulated for their careful use of this ELISA that resulted in this discovery. This paper is unconventional in that much of it is devoted to methodological troubleshooting rather than unfolding a "story" of biomedical discovery. Nevertheless, because the ELISA being reported on was previously published (and in rather high profile journals as the authors point out), it makes for a valuable case study of how to contend with the lack of specificity in an antibody-based assay. Moreover, the workaround developed by the authors to achieve a modest degree of specificity in the assay combined with application of the assay to AAA patient plasma samples with the finding that MPO-DNA complexes are not correlated with other known markers of neutrophil activation or NET formation make it a story worth reporting in the scientific literature. Prior to publication, however, numerous issues need to be addressed by the authors. Since one of these involves re-analysis of the MPO-DNA complex ELISA data from the AAA patients, a "major" revision was recommended. But barring any unforeseen problems, this reviewer feels that once the specific issues below are addressed, the manuscript should be published.

Specific Comments:
1) Methods; Fab fragment preparation: Additional technical details are needed regarding this separation so that readers can repeat it themselves if desired. For example, with the description given, it's impossible to determine how, exactly, fractions were collected for the data in Fig. 4.
Reply: We would like to thank the reviewer for the profound review of our work. More details are now provided on Fab fragment preparation in the respective Methods section (line 190).

2) Table 1: The number of significant figures reported for many entries is too high. Most likely only 2-3 significant figures are warranted.
Reply: Table 1 summarizes all assays performed with the respective calibrator batches with the initial MPO-DNA ELISA protocol. Please note that the number of assays conducted with control supernatant from unstimulated neutrophils was much lower than for calibrator derived from PMA-treated neutrophils, since control supernatant consistently gave signals close to background while calibrator from stimulated neutrophils was applied in all assays. To enable a direct comparison of OD values derived from control and PMA-treated samples assayed on the same plates, we have added Suppl. S1 Table to the manuscript (as referenced in the manuscript text on line 333).

3) The data in Table 1 are derived from three different batches of neutrophils from two different donors. Please clarify which donor(s) contributed to which batches.
Reply: We clarified this point by adding the respective information to the legend of Table 1 (line 328).

4) Lines 436-438 states, "Similar results were found for application of a commercially available blocker (The Blocking Solution, S2 Fig E-F)." The results in panel F are not at all similar to the results in panel D. In fact, these results seem to show in vivo specificity that is at least as good as the final solution worked out and presented in Fig. 8D and in Fig. S3. Why was this apparent finding seemingly disregarded? In a related matter, lines 695-697 state, "As this protocol modification [seen in Fig. 8D and Fig. S3D] was the only measure that improved the ELISA specificity for MPO-DNA complex detection in plasma samples, it was finally applied for investigation of clinical AAA samples." Please also adjust this statement as needed in light of this critique.
Reply: We would like to draw the reviewer's attention to the fact that in S2 Fig panels E and F the application of "The Blocking Solution" led to a massive reduction of the ELISA signal achieved with MPO capture antibody (as did the application of LCBM, LCB and LCBS in panels C and D) when compared with the signal achieved with the initial ELISA protocol (IEP). Moreover, upon application of "The Blocking Solution" the signals of MPO antibody and isotype antibody coated wells again gave similar ODs (at a very low level) thus yielding no improvement of detection window between MPO and isotype coated wells - as opposed to the experiments depicted in Fig. 8D and in Fig. S3D (please note that Fig S3D has been changed to Fig. 8E in the revised manuscript). We have tried to phrase this finding more explicitly in the manuscript text (line 479).

5) Fig. 4: Indicate the band corresponding to antibody light chain in lane 2.
Reply: We thank the reviewer for calling our attention to this missing information. We have modified the labels in Figures 4b and 4c and adapted the figure legend accordingly (line 509).

6) Line 483: There are no uncoated wells in panel B of Fig. 5. The statement in line 483 should refer only to panel C.
Reply: We have corrected the reference to Fig. 5B and 5C (line 527).

7) Line 498: Please refer to concentrations in the same terms in which they are displayed in Fig. 6. (Dilution factors are mentioned in the text but "relative concentration" is used in the figure.)
Reply: We have rephrased the main text (line 542) as well as the respective figure legend (line 550) to enable a better understanding. Please note that the stated dilution factors (1:4 and 1:100) refer to plasma samples, while the stated relative concentrations refer to the amount of added calibrator.

8) For the final modified assay with moderate specificity that was developed, nonspecific signals are subtracted from specific signals providing a net signal that is used for relative quantification and comparison to other samples. (This is possible because of the isotype control that is run for each sample.) The problem with this approach is that the calibrator curves are non-linear. This means that the same net signal excerpted from a low relative concentration segment in the calibration curve will correspond to a greater concentration difference (between actual sample and its isotype control) than the same net signal excerpted from a high relative concentration segment on the calibration curve. The magnitude of this discrepancy may have influenced the conclusions drawn on the AAA patient samples. The data that went into Table 2 should be re-processed and re-analyzed after taking this issue into account.
Reply: Since the calibrator represents the specific MPO-DNA signal (i.e. was consistently negative for isotype coated wells), we opted for the variant to first subtract the unspecific plasma signal of isotype controls from the presumably combined (specific and unspecific) signal in MPO-coated wells before calculating MPO-DNA complex concentrations in reference to the calibrator. To address the reviewer´s concern we have included alternative calculation methods into the manuscript (line 654 and Supplementary Figure S4). In addition, measurements were performed with both, the initial and the final ELISA protocol.

Please note that based on the suggestion of reviewer #5 human plasma samples from an endotoxemia model are now more prominently featured in the manuscript than the AAA patient samples. This is why we have conducted the comparison of calculation methods for the new sample set: Unspecific isotype signals were either disregarded (S4 Fig C) or were deducted from recorded MPO-DNA values before (S4 Fig A) or after (S4 Fig B) calculation of relative units. While the results differed moderately, no significant positive correlations with other plasma parameters of neutrophil activation or NET formation were recorded for any of the approaches.

9) Lines 578-587: How many intra-assay sets were analyzed for both calibrators and controls? Also, please explain in greater detail exactly how multiple inter-assay variability experiments were conducted? Typically just one value is reported for this parameter. Also, explain in greater detail how control plasma samples were included on each single plate to adjust for high inter-assay variation. How did this adjustment work, exactly? Inter-assay variation on the order of 30% is quite high and is often considered unacceptable for certain types of measurements. Readers should be cautioned about this issue in the Discussion.
Reply: In reference to the reviewer´s questions we have included the following pieces of information in

- Results - Establishment of a modified MPO-DNA ELISA for detection of MPO-DNA complexes in human plasma (line 632): With the aim of determining the intra- and interassay coefficients of variation (CV) for this modified MPO-DNA complex ELISA protocol six calibrator concentrations and three plasma samples were applied in duplicates to six consecutive ELISA plates. The mean intra- and interassay CV values for calibrator samples ranged at 9.3% and 12.7%, whereas plasma samples exhibited coefficients of 16.0% and 29.0%, respectively.
- Methods - Final (modified) MPO-DNA complex ELISA protocol (line 249): To adjust for high interassay variation, three control plasma samples were applied to each plate in the endotoxemia and AAA sample analysis. The calculated MPO-DNA levels of the controls were set in reference to the initially determined values of the first plate. Thus, an averaged “conversion factor” was determined and subsequently applied to all plasma measurements of the respective plate.
- Discussion (line 793): As this protocol modification was the only measure that improved the ELISA specificity for MPO-DNA complex detection in plasma samples, it was finally applied for investigation of human blood samples of acute and chronic inflammatory conditions despite the circumstance that intra- and interassay CVs were as high as 16% and 29%, respectively.

10) Line 614: For the high impact publications cited here, point out which ones did not use isotype controls.
Reply: We added the requested information to the manuscript in the Discussion section (line 695).

11) This reviewer thinks that Fig. S3 (or at least panels D-G within it) should be part of the main text and not in supplemental information.
Reply: We have followed the reviewer’s suggestion and have inserted S3 Fig D and two of the three panels of S3 Fig E-G into figure 8. The main manuscript text (line 627) as well as the respective figure legends (lines 609 and 1065) were adapted accordingly.

12) Line 966: Describing the data in Fig. S2 panel F as “1:5 diluted plasma samples using various buffers for blocking” is too vague. What is meant by "using various buffers for blocking"? There appears to be only one blocking buffer (or buffer combination) used in this experiment. Also, what was the antibody dilution in this experiment?
Reply: The respective pieces of information are now included in the legend to Supplementary Figure S2 (lines 1044 and 1046).

13) Please adjust the caption to Fig. 3 to better explain exactly where the isotype control data points and lines are (i.e., exactly where they are hidden in the figure).
Reply: We have added the missing information to the respective figure legend (line 453).

14) Fig. 7: Please explain why the same dilution of the same donor plasma doesn't give the same (or similar) OD in the different graphs here. Is this simply due to the poor inter-assay precision?
Reply: We have corrected the label of Fig 7B, i.e. “donor 2” was replaced by “donor 3”, as the second donor in this very panel is indeed different from donor 2 in Fig 7A and Fig 7C – which eliminates the seeming discrepancy for this donor. However, the donor 1 sample is consistent for all three panels: While the 1:10 plasma dilution gave similar ODs in the assays presented in Fig 7A and B, the recorded signal for 1:100 diluted plasma differed substantially between assays of Fig 7B and C. This high interassay variability in ODs is indeed another limitation of the original MPO-DNA ELISA protocol (largely attributed to the unspecific reaction). Yet, we noted that relative signal levels were reproducible when we repeatedly and concomitantly measured the same samples from distinct plasma donors (as is now also mentioned in the Results section, line 378).

Reviewer #4:
The authors improved a published MPO-DNA ELISA method for plasma samples, since it showed low specificity for in vivo plasma samples. I think the work is interesting and meaningful. However, some issues should be presented clearer.

How author confirm the existed method was inaccurate? Is there any testing method for MPO-DNA to verified your opinion?
Reply: Our finding that the MPO-DNA ELISA lacks specificity for NET detection in plasma samples is consistently based on the comparable signal of isotype control and MPO capture antibody. Unfortunately, there are no reliable alternatives to determine the circulating MPO-DNA complexes in blood samples. As performed by a few research groups we attempted to detect DNA attached to MPO by means of a fluorescent, intercalating dye as opposed to the anti-DNA antibody. However, in our hands, this approach was not sufficiently sensitive for MPO-DNA measurement in human plasma. A comparable statement has now been included in the Discussion section (line 698).

In result section, I think the process of improvement could be concentrated. And more optimization of new method and comparison between new and published methods could be discussed.
Reply: We agree with the reviewer that the manuscript is rather lengthy. But given the sensitive issue of questioning previously published data which were based on this assay, we considered it necessary (and hope the reviewer will concur) to document the attempts and failures of assay improvement in much detail.
In line, we have adopted the reviewer´s suggestion to include a direct comparison between the original and the final ELISA protocol which is now shown for the new sample set of human endotoxemia. Please refer to the Results section (line 645), Figure 9, Supplementary Figure S4 and the Discussion section (line 803).

A conclusion section is needed.
Reply: We have restructured the Discussion and introduced a Conclusion section (line 817).

Reviewer #5:
In this manuscript, the authors present data on the assay on NETs using a coupled immunoassay. While this assay performed well with in vitro generated NETs, a number of technical issues were encountered using blood samples.
The authors explore in detail possible explanations for the technical issues and provide an approach to improve the assay.

The results are perhaps surprising in view of the many publications on the subject, raising questions as to why the problems were not previously detected.

One concern relates to the number of donors studied as well as the study of patients with abdominal aortic aneurysms. While patients with AAA may have NETs, it is hard to know how these values would compare with patients with infectious or autoimmune disease. I think therefore that it is important to study in the original as well as revised assay a larger panel of both control samples as well as samples with a broader range of autoimmune/inflammatory/infectious diseases. Since the literature on the latter conditions is likely larger than that on AAA, the value of the paper would be increased by allowing comparison with the current literature.
Given the implications of the study for the assay of NETs, additional analysis of samples is important.
Reply: We appreciate the reviewer´s concern that circulating NET parameters in AAA patient samples might not be comparable to levels achieved during infectious or autoimmune conditions and hence inclusion of additional clinical samples is warranted. Unfortunately, we do not have ready access to such sample sets. However, we have previously participated in a human endotoxemia study (based on a model of low-dose LPS infusion into healthy volunteers: Methods, line 255) which offered us the possibility to analyze MPO-DNA complexes by the original and modified ELISA protocol – along with other markers of neutrophil activation and NET formation – in healthy volunteers at baseline and during a time course of induced acute inflammation triggered by experimental endotoxemia. The results, as specified in the manuscript text (lines 639 and 798), Figure 9, Supplemental Figure S4 as well as Table 2, illustrate an impressive rise in the circulating NET parameter citH3 and in DNA-histone complexes peaking at 4 h after LPS challenge. As previously observed for the AAA cohort, strong positive correlations were detected for all neutrophil activation and NET parameters except for MPO-DNA complexes. Hence, with this additional sample set we hope to have provided further convincing evidence for the limited applicability of the MPO-DNA ELISA for NETs formed *in vivo*.

We thank all the reviewers for their valued contribution and hope to have adequately addressed their concerns by the introduced changes to improve the quality of our manuscript. We thus hope to meet the quality standards for publication in *PLOS ONE* and we look forward to hearing from you.

Yours sincerely,

Christine Brostjan

Christine Brostjan
